# Influence of an Infectious Diseases Specialist on ICU Multidisciplinary Rounds

**DOI:** 10.1155/2014/307817

**Published:** 2014-04-17

**Authors:** David N. Gilbert

**Affiliations:** Department of Medical Education, Providence Portland Medical Center, 5050 NE Hoyt, Suite 540, Portland, OR 97213, USA

## Abstract

*Objective*. To ascertain the influence of a physician infectious diseases specialist (IDS) on antibiotic use in a medical/surgical intensive care unit. *Method*. Over a 5-month period, the antibiotic regimens ordered by the ICU multidisciplinary team were studied. The days of antibiotic therapy (DOT) when management decisions included an IDS were compared to DOT in the absence of an IDS. The associated treatment expense was calculated. *Results*. Prior to multidisciplinary rounds (MDRs), 79-80% of the patients were receiving one or more antibiotic. IDS participation occurred in 61 multidisciplinary rounding sessions. There were 384 patients who before MDRs had orders for 669 days of antimicrobial therapy (DOT). After MDRs, the antimicrobial DOT were reduced to 511 with a concomitant cost saving of $3772. There were 51 MDR sessions that occurred in the absence of the IDS. There were 352 patients who before MDRs had orders for 593 DOT. After MDRs, the DOT were reduced to 572 with a cost savings of $727. The results were normalized by number of patients evaluated with statistically greater reductions when MDRs included the IDS. In addition, the number of rounding sessions with a reduction in DOT was greater with the participation of the IDS. *Conclusion*. The addition of an IDS to multidisciplinary ICU patient rounds resulted in a reduction in antibiotic DOT and attendant drug expense.

## 1. Introduction

In point prevalence studies, roughly two-thirds of patients in medical or medical/surgical intensive care units (ICUs) are administered antimicrobial therapy [1]. Roughly seven years ago, as part of our institution's multifaceted Antimicrobial Stewardship Program, it was postulated that inclusion of an infectious diseases specialist (IDS) as part of multidisciplinary rounds (MDRs) could favorably influence the use of antimicrobials. After several years, the general consensus was that the inclusion of an IDS was beneficial to patient care. It was decided to document the influence of the IDS as best as possible with limited human resources. Hence, only a few endpoints are reported in the study herein described. On the other hand, the data are relevant to a general community teaching hospital with sufficient IDS resources to allow frequent participation in MDRs.

## 2. Materials and Methods

### 2.1. Multidisciplinary Rounds

PPMC has 483 licensed beds and is a community teaching hospital with an internal medicine residency. PPMC is not a trauma center. The ICU is a combined medical/surgical ICU that is separate from the medical/surgical coronary care units. The ICU has 23 beds, but the multidisciplinary team follows a maximum of 15 patients. The basic team consists of 1 of 11 pulmonary critical care physicians, 1 of 4 critical care pharmacists, 2-3 internal medicine residents, and the patient's nurse. The critical care physician and the ICU pharmacist rotate weekly. The residents rotate every four weeks. Other physician specialists are included, as needed, on a case-by-case basis, for example, neurology, general surgery, and so forth.

PPMC is served by six IDSs. Only one (the author) of the six participates in MDRs. The other five IDSs provide consultations throughout the medical center plus leading institution-wide programs in Infectious Diseases Antibiotic Stewardship and Infection Control. On occasion, a hospitalized infectious diseases consultation patient will be transferred into the ICU and, hence, would receive care from two infectious diseases physicians. The hospital Antimicrobial Stewardship Program monitors antimicrobial use outside of the ICU while the ICU stewardship responsibility is borne primarily by the IDS on the MDR team.

The IDS, barring conflicts, participated in MDRs daily from 8 a.m. to 10-11 a.m., Monday through Friday. The IDS contributes to the team activity in many ways: expanding and refining the differential diagnosis, advising on diagnostic maneuvers, interpretation of results of infectious diseases-related tests, suggested empiric and/or specific and/or prophylactic antimicrobials, addressing adverse drug reaction issues, and providing advice on isolation procedures. There was no attempt to measure antimicrobial DOT subsequent to discharge from the ICU.

### 2.2. Study Procedure

The other IDSs at PPMC are overcommitted and had no time to help with study implementation. There are no Infectious Diseases fellows at PPMC. The IDS assigned to ICU MDRs has a host of other teaching and infrastructure activities. Hence, we were forced to select simple, achievable endpoints to assess the influence of the IDS in MDRs.

We selected antimicrobial days of therapy (DOT). New patient admissions are often started on multiple empiric antimicrobials concomitant with diagnostic tests designed to clarify the disease process. Several hours may pass before the patient is presented to the MDR team at which time the antimicrobial therapy may be adjusted. It was postulated that the influence of the IDS expertise could be assessed by the difference in the projected total DOT of all the patients in the ICU at the start of rounds versus the projected DOT prescribed after discussion at MDRs. The total DOT might increase, decrease, or remain unchanged.

As a control, we measured the antimicrobial days of therapy on days the IDS was not present, for example, weekends, holidays, meetings, and so forth. On such days, the assigned ICU pharmacist collected the data. There were 39 days that the IDS was absent and the ICU pharmacist failed to collect a complete database; the days with incomplete data were excluded from the data analysis.

The number of antimicrobial days of therapy (DOT) was determined for each rounding session. Antibacterials, antifungals, and antivirals (with the exception of antiretrovirals) were included. We selected DOT as a reasonable tool to document the daily prerounds and postrounds use of antimicrobials. We could have used defined daily doses (DDD) as is popular in some countries. Based on the publications of Polk and colleagues, we elected to use DOT.

Polk et al. define a DOT as a single dose or multiple doses of a given drug administered within 24 hrs. Hence, a single dose of ceftriaxone or six doses of nafcillin are both one DOT. In short, for each rounding session, we obtained a total DOT before rounds and again after rounds.

### 2.3. Study Period

We arbitrarily selected January 1, 2013, to June 1, 2013, as the study period. The data reflect the influence of one IDS who is the author of the paper.

### 2.4. Cost of Antimicrobials

The pharmacy buyer for PPMC compiled a comprehensive list of the cost of all the antimicrobials administered to the patients enrolled. The costs were acquisition costs. For the expense calculation, we projected the cost of 24 hours of therapy for all drugs prescribed based on the prerounds and postrounds planned regimens. The expense of diluents and labor of nurses and pharmacists were not evaluated.

### 2.5. Statistics

Robust regression was used for comparison of days of antimicrobial therapy per patient and comparison of cost of therapy per patient.

Fischer's Exact Test was used to compare the number of rounding sessions that resulted in an increase in antibiotic days of therapy for all patients seen by the ICU team in that session versus an overall decrease in days of therapy versus no change in antibiotic days of therapy.

## 3. Results

The average percentage of ICU patients receiving antibiotics at the start of the rounding sessions was 79% when the IDS was present and 80% when the IDS was absent. The results that follow reflect decisions on antimicrobial therapy of both those patients who were receiving an antimicrobial regimen at the start of the rounding session and those patients whose antimicrobial therapy was initiated during MDRs. The daily patient census varied from a low of 3 to a high of 13; the average was 7 patients with no difference between the presence and absence of the IDS.

The total number of rounding sessions, number of patients receiving antimicrobials, and the cost of the treatment regimens are summarized in Table 1. The results indicate less antimicrobial use when the IDS was present for MDRs. The totals were divided by the number of patients evaluated and subjected to statistical analysis.

The IDS participated in 61 multidisciplinary rounding (MDRs) sessions. There were 384 patients who before rounds had orders for 669 antibiotic DOT. After MDRs, the DOT were reduced to 511 with a concomitant cost saving of $3772. The IDS did not participate in 51 MDRs. There were 352 patients who before rounds had orders for 593 DOT. After MDRs, the DOT decreased to 572 DOT with a cost saving of $727. The reduction in DOT with IDS participation was normalized to DOT per patient and achieved statistical significance; *P* = 0.001.

In addition, the number of rounding sessions where the DOT were either increased, decreased, or unchanged was determined (Table 1). The number of rounding sessions that resulted in an overall group decrease in DOT was statistically greater with participation of the IDS; *P* = 0.001.

## 4. Discussion

In patients admitted to ICUs, it is hoped that a daily assessment of the overall clinical picture and microbiologic results will allow the multidisciplinary team to deescalate empiric antibiotic therapy. It takes confidence to deescalate or discontinue antimicrobial therapy. An IDS embedded in the multidisciplinary team is a major asset in interpreting clinical signs and symptoms, epidemiology, laboratory and imaging data, adverse drug reactions, and culture data. In addition to the educational value, the result is a reduction in the insecurity inherent in treatment decisions. Our data demonstrate a decrease in antibiotic days of therapy whether an infectious diseases specialist was present or not. However, the magnitude of the decrease was substantively larger when an IDS was present. As expected, the antibiotic dollar cost per patient was also significantly lower. The per patient data may better reflect antibiotic stewardship than the DOT for the total of all patients present on a given day.

A literature review failed to identify other institutions that have used the approach herein described. The literature indicates a variety of approaches to antimicrobial stewardship in the ICU. In a review published in 2011, 24 studies were identified [2]. Two additional pertinent studies were published in 2013 [3, 4]. The ICU types varied: medical, surgical, trauma, pediatric, and various combinations thereof. The number of patients studied varied widely: from 61 to 13,293. The most common study design was an assessment of any changes in antimicrobial use before and after an intervention (20 of the 26 studies). Six general types of intervention were identified:

1. antibiotic restriction or preapproval (six studies)
2. formal infectious diseases consultation (five studies)
3. deescalation protocol implementation (two studies)
4. guideline for prophylaxis or treatment (two studies)
5. formal reassessment of antibiotics on a prespecified day of therapy (four studies)
6. daily prompting as to need for empiric antibiotic therapy (one study)
7. computer-assisted decision support (six studies).

To various degrees, all studies were beneficial as measured by decreased use of antimicrobials and concomitant reduced expense, shorter course of therapy, less inappropriate therapy, and fewer adverse events.

In the total of 26 studies identified, an IDS was embedded in ICU MDRs in ICUs in Argentina, Brazil, and perhaps in Hungary [5–7]. The interventions varied from 6 months to 2 years. The positive influence of the presence of an IDS was determined using before and after model. The other 23 studies utilized interventions with a lower intensity of interaction, for example, periodic consultations, specific patient discussions, and chart reviews [1, 2]. Only three randomized controlled trials were identified, and they did not focus on the influence of IDS as part of MDRs. Two studies randomized patients with pneumonia to standard or shorter course of therapy of pneumonia; one assessed the influence of a computerized antibiotic guide [8–10].

Our data provides robust support for the value of the permanent addition of an IDS to the multidisciplinary team of a medical-surgical intensive care unit. Although not perfect, the reduced use of antimicrobials provides strong statistical support for this approach. The potential for improved quality of care is clear. With 80 percent of ICU patients administered one or more antibiotics, it is likely that, on a given day, more patients are exposed to an antibiotic than are requiring mechanical ventilation.

Our study has several weaknesses. Our approach requires an institutional willingness to provide salary support for the time of the IDS. Future studies will assess the comparative cost effectiveness of ongoing daily involvement versus the “in and out” approaches described in the literature.

Another advantage of daily participation is continuity of care. Albeit hard to quantify, immersion in the subtleties of the patient's illness clarifies the database and improves treatment decisions.

This is a single center study evaluating the influence of a single IDS. There were not sufficient resources to catalog patient demographics, comorbidities, specific infection syndromes, etiologic bacteria, outcome of therapy, or impact on resistance. The study was not designed to ascertain the influence of antibiotic deescalation on resolution of the infectious disease under treatment, number of ventilator days or ICU days, or reduced short-term mortality. We did not enumerate the influence of reduced antibiotic days on drug-related adverse effects. The IDS was present for only 2-3 hrs. per day. The antibiotics administered to the patient often change over the ensuing 22 hours. The impact of the IDS might be augmented by a late afternoon brief reassessment of selected patients. Further, the availability of the IDS was not randomly determined. In short, despite these many limitations, the data provide insight into the potential influence of an embedded IDS on antimicrobial use in the ICU. Future studies are indicated and should address the limitations mentioned.

In summary, our data support the inclusion of an IDS as part of the ICU MDRs. Directly measurable benefits are fewer days of antibiotic therapy and attendant reduced drug expense. Theoretical benefits are a reduced risk of selection of resistant bacteria, fewer drug-related adverse effects, and assistance in clarification of the clinical illness, and the opportunity to teach in the course of patient care.

## Figures and Tables

**Table 1:** Influence of the presence and absence of an infectious diseases specialist (IDS) on antibiotic use decisions by the ICU Multidisciplinary Team at Providence Portland Medical Center.

	In the presence of IDS	In the absence of IDS
Number of rounding sessions	61	51
Total number of patients receiving antimicrobials pre-rounds	384	352
Total days of therapy (DOT)		
Prerounds	669	593
Postrounds	**511**	**572**
Difference	−158	−21
Decrease DOT/patient	−0.41*	−0.06*
Total cost of antimicrobial regimen, $		
Prerounds	17,521	15,153
Postrounds	**13,749**	**14,426**
Difference	−3,772	−727
Decrease cost/patient	−9.82*	−2.10*
Number of multidisciplinary rounds where DOT		
Increased	0	14
Did not change	15	14
Decreased	46*	23*

*P = 0.001.
